# Functional Genetic Variant of Long Pentraxin 3 Gene Is Associated With Clinical Aspects of Oral Cancer in Male Patients

**DOI:** 10.3389/fonc.2019.00581

**Published:** 2019-07-03

**Authors:** Chia-Ming Yeh, Chiao-Wen Lin, Chun-Yi Chuang, Yu-Fan Liu, Chia-Hsuan Chou, Shun-Fa Yang, Mu-Kuan Chen

**Affiliations:** ^1^Institute of Medicine, Chung Shan Medical University, Taichung, Taiwan; ^2^Cancer Research Center, Changhua Christian Hospital, Changhua, Taiwan; ^3^Department of Otorhinolaryngology-Head and Neck Surgery, Changhua Christian Hospital, Changhua, Taiwan; ^4^Institute of Oral Sciences, Chung Shan Medical University, Taichung, Taiwan; ^5^Department of Dentistry, Chung Shan Medical University Hospital, Taichung, Taiwan; ^6^School of Medicine, Chung Shan Medical University, Taichung, Taiwan; ^7^Department of Otolaryngology, Chung Shan Medical University Hospital, Taichung, Taiwan; ^8^Department of Biomedical Sciences, College of Medicine Sciences and Technology, Chung Shan Medical University, Taichung, Taiwan; ^9^Department of Medical Research, Chung Shan Medical University Hospital, Taichung, Taiwan

**Keywords:** long pentraxin 3, oral squamous cell carcinoma, single-nucleotide variation, metastasis, splicing, tumor progression

## Abstract

Long pentraxin 3 (PTX3) is produced by various cell types and is correlated with tumor progression in various tumor types. However, the clinical significance of *PTX3* polymorphisms in oral cancer and their correlation with the risk of cancer are still unclear. In this study, we assessed the influence of *PTX3* gene polymorphisms and environmental factors on susceptibility to oral tumorigenesis. We recruited 865 cases with oral cancer and 1,189 controls. Four single-nucleotide variations of the *PTX3* gene (rs1840680, rs2305619, rs3816527, and rs2120243) were verified using a real-time polymerase chain reaction in control participants and cases with oral cancer. We found that rs3816527 in smokers was correlated with the development of late-stage cancer (odds ratio [OR], 2.328; 95% confidence interval [CI], 1.078–5.027) and increased lymph node metastasis (OR, 2.152; 95% CI, 1.047–4.422). Moreover, additional bioinformatics analysis results showed that the rs3816527 C allele variant to the A allele exhibited the strongest exonic splicing enhancer activity. In conclusion, our results suggested that *PTX3* rs3816527 plays a role in oral cancer development.

## Introduction

The incidence of oral cancer in Asia and more particularly Taiwan increases annually. In addition to tobacco smoking and alcohol consumption, betel quid chewing is a major cause of oral cancer (1–4). Moreover, the annual number of deaths due to oral cancer among men is increasing rapidly (5). The global 5 year mortality rate of oral cancer is ~50%. Although considerable progress has been made in surgery, chemotherapy, and radiotherapy, no considerable improvements have been made in the preceding 50 years (6).

Pentraxins (PTXs) are a superfamily of conserved proteins that contain the pentraxin domain. Proteins of the pentraxin family are multifunctional and participate in acute immunological responses (7). PTX3, also known as TGF14, is a transmembrane molecule expressed in a variety of human tissues (8, 9). PTX3 is secreted by natural immune cells in response to inflammatory cytokines, such as interleukin-1 β (IL-1β) and tumor necrosis factor α (TNFα), or selected pathogen-associated molecular patterns (9–11). Therefore, *PTX3* may play a vital role at the crossroads of inflammation increase (12, 13), innate immunity (14–16), tissue repair stimulation (17, 18), and cancer (19–21). *PTX3* promotes cell migration and invasion in several cancers, and the expression of *PTX3* correlates with tumor progression in various human tumor types (19, 22–24).

Genetic variations contribute to susceptibility to common diseases such as cardiovascular disease, diabetes, inflammatory disease, and cancer (25–29). Single-nucleotide variations (SNVs) may be a causative genetic variant that can affect the expression and structure of proteins, thereby directly contributing to disease (30). The *PTX3* gene is located on chromosome 3 and contains three exons and two introns. Previous studies have described that SNVs in *PTX3* (rs2305619 and rs1840680) have functional significance. Results indicated that the A allele of *PTX3* SNVs (rs2305619 and rs1840680) is associated with higher plasma levels of PTX3 (31, 32). In addition, the A alleles of rs2305619 and rs1840680 are associated with susceptibility to *Pseudomonas aeruginosa* and *Mycobacterium tuberculosis* infections (33, 34). Carmo et al. also revealed that genetic variations in *PTX3* (rs2305619 and rs1840680) and plasma levels were associated with hepatocellular carcinoma (35). Moreover, Hakelius et al. indicated that PTX3 played a major role in non-malignant and oral cancer malignant disease processes (36). However, few studies have investigated the association of *PTX3* polymorphisms in oral cancer. Therefore, we investigated the relationship between four *PTX3* gene polymorphisms (rs1840680, rs2305619, rs3816527, and rs2120243; Table 1) and clinicopathological characteristics of patients with oral cancer to identify those with an increased risk of oral cancer.

**Table 1:** Information on *PTX3* SNPs.

**Variable**	**rs2120243**	**rs2305619**	**rs3816527**	**rs1840680**
Chromosome	3:157429779	3:157437072	3:157437525	3:157438240
Exon	–	–	2	–
Nucleotide change	A>C	A>G	C>A	A>G
mRNA position	–	–	286	–
Function	TFBS	Intron variant	Non-synonymous	Intron variant
dbSNP allele	–	–	GCC ⇒ GAC	–
Protein residue	–	–	A [Ala] ⇒ D [Asp]	–
Allele frequencies	0.6744	0.6358	0.7845	0.6738

*Allele frequencies were obtained from population databases gnomAD (https://gnomad.broadinstitute.org/) for East Asian population*.

## Materials and Methods

### Study Population

The participants of this case–control study were 865 male cases with oral cancer of squamous cell carcinoma recruited from Changhua Christian Hospital in Changhua and Chung Shan Medical University Hospital in Taichung, Taiwan, between 2007 and 2017, and 1,189 cancer-free male controls selected from the Taiwan Biobank. The oral cancers of squamous cell carcinoma in this study were pre-specified to include any cancers that originated from buccal mucosa (*n* = 314; 36.3%), tongue (*n* = 268; 31.0%), gingiva (*n* = 78; 9.0%), palate (*n* = 40; 4.6%), floor of the mouth (*n* = 25; 2.9%), and other areas (*n* = 140; 16.2%). The Institutional Review Board of Chung Shan Medical University approved this study (CSMUH No: CS13214-1). All participants provided written informed consent. Personal characteristics and information, including demographic characteristics; tobacco smoking, betel quid chewing, and alcohol consumption habits; and the medical histories of the participants, were investigated using interviewer-administered questionnaires.

### Determination of Genotypes

Genomic DNA from peripheral blood leukocytes was extracted using a QIAamp DNA Blood Mini Kit (Qiagen, Valencia, CA, USA) following the manufacturer's protocol (37). DNA was dissolved in ethylenediaminetetraacetic acid (EDTA) buffer (10 mM Tris, 1 mM EDTA; pH 7.8) and then quantified by measuring absorbance at 260 nm. Finally, the preparation was stored in a −20°C refrigerator and later analyzed using a real-time polymerase chain reaction (PCR) system. Allelic discrimination for the PTX3 SNV was performed using a TaqMan assay (ID C_12069244_10 for rs1840680, C_22275654_10 for rs2305619, C_3035766_30 for rs3816527, and C_11796613_20 for rs2120243) with an ABI StepOne Real-Time PCR System (Applied Biosystems). The genotypic frequencies of *PTX3* were further evaluated using the SDS v3.0 software program.

### Bioinformatics Analysis

Several bioinformatics tools were used to assess the putative functional relevance of the rs3816527 PTX3 polymorphism. SNPinfo was used to predict the function of the rs351855 polymorphism. Splicing enhancement activity was analyzed using ESEfinder. We used the National Center for Biotechnology Information (NCBI) database to determine splicing types of PTX3. Furthermore, protein structure homology modeling of PTX3 was performed using SWISS-MODEL.

### Statistical Analysis

The Mann–Whitney *U*-test was used to compare differences in demographic characteristics between healthy controls and cases with oral cancer. Multiple logistic regression models were used to determine the association between genotypic frequencies and oral cancer risk after adjustment for age, betel quid chewing, cigarette smoking, and alcohol consumption. Data were analyzed using SAS 9.1 statistical software. A *p* <0.05 was considered significant.

## Results

### Demographic Characteristics of the Participants

Table 2 displays the results of the statistical analysis of the participants' demographic characteristics. A total of 2,054 participants were enrolled, namely 865 male cases with oral cancer and 1,189 male controls. Results revealed significant differences in cigarette smoking (*p* < 0.001), betel quid chewing (*p* < 0.001), and alcohol consumption (*p* < 0.001) between the cases with oral cancer and the controls.

**Table 2:** Distribution of demographic characteristics in 1,189 controls and 865 male individuals with oral cancer.

**Variable**	**Controls (*N* = 1,189)**	**Cases (*N* = 865)**	***p*-value**
**AGE (YRS)**			
≦55	607 (51.0%)	454 (52.5%)	*p* = 0.521
>55	582 (49.0%)	411 (47.5%)	
**BETEL QUID CHEWING**			
No	991 (83.4%)	188 (21.7%)	*p* <0.001^*^
Yes	198 (16.6%)	677 (78.3%)	
**CIGARETTE SMOKING**			
No	558 (46.9%)	99 (11.5%)	*p* <0.001^*^
Yes	631 (53.1%)	766 (88.5%)	
**ALCOHOL DRINKING**			
No	954 (80.2%)	414 (47.9%)	*p* <0.001^*^
Yes	235 (19.8%)	451 (52.1%)	
**STAGE**			
I+II		431 (49.8%)	
III+IV		434 (50.2%)	
**TUMOR T STATUS**			
T1+T2		496 (57.3%)	
T3+T4		369 (42.7%)	
**LYMPH NODE STATUS**			
N0		583 (67.4%)	
N1+N2+N3		282 (32.6%)	
**METASTASIS**			
M0		856 (99.0%)	
M1		9 (1.0%)	
**CELL DIFFERENTIATION**			
Well-differentiated		119 (13.8%)	
Moderately or poorly differentiated		746 (86.2%)	

*Mann–Whitney U-test or Fisher's exact test was used between healthy controls and individuals with oral cancer*.

* *p <0.05 was considered statistically significant*.

### *PTX3* Gene Polymorphisms in Cases With Oral Cancer and Controls

To investigate the association between *PTX3* gene polymorphisms and oral cancer risk, the genotypic, and allelic frequencies of *PTX3* in the individuals with oral cancer and the controls were established in this investigation (Table 3). After adjustment for betel quid chewing, cigarette smoking, and alcohol consumption, no significant difference was observed between the participants with oral cancer who had rs1840680, rs2305619, rs3816527, and rs2120243 polymorphisms of the *PTX3* gene and those with wild-type (WT) genes.

**Table 3:** Genotyping and allelic frequency of *PTX3* single-nucleotide variations (SNVs) in individuals with oral cancer and controls.

**Variable**	**Controls (*N* = 1,189 (%))**	**Cases (*N* = 865 (%))**	**OR (95% CI)**	**AOR (95% CI)**
**rs1840680**				
GG	531 (44.7%)	375 (43.4%)	1.000 (reference)	1.000 (reference)
GA	532 (44.7%)	407 (47.0%)	1.083 (0.901–1.303)	1.056 (0.832–1.341)
AA	126 (10.6%)	83 (9.6%)	0.933 (0.686–1.268)	0.876 (0.591–1.297)
GA+AA	658 (55.3%)	490 (56.6%)	1.054 (0.884–1.258)	1.020 (0.812–1.281)
G allele	1594 (67.0%)	1157 (66.9%)	1.000 (reference)	1.000 (reference)
A allele	784 (33.0%)	573 (33.1%)	1.007 (0.883–1.149)	0.978 (0.825–1.159)
**rs2305619**				
GG	493 (41.5%)	346 (40.0%)	1.000 (reference)	1.000 (reference)
GA	550 (46.3%)	428 (49.5%)	1.109 (0.920–1.336)	1.126 (0.885–1.433)
AA	146 (12.2%)	91 (10.5%)	0.888 (0.661–1.194)	0.786 (0.537–1.151)
GA + AA	696 (58.5%)	519 (60.0%)	1.062 (0.889–1.270)	1.050 (0.835–1.322)
G allele	1536 (64.6%)	1120 (64.7%)	1.000 (reference)	1.000 (reference)
A allele	842 (35.4%)	610 (35.3%)	0.994 (0.873–1.131)	0.960 (0.812–1.135)
**rs3816527**				
AA	734 (61.7%)	511 (59.1%)	1.000 (reference)	1.000 (reference)
AC	402 (33.8%)	317 (36.6%)	1.133 (0.941–1.364)	1.191 (0.937–1.515)
CC	53 (4.5%)	37 (4.3%)	1.003 (0.649–1.549)	1.110 (0.633–1.947)
AC + CC	455 (38.3%)	354 (40.9%)	1.118 (0.934–1.337)	1.182 (0.938–1.490)
A allele	1870 (78.6%)	1339 (77.4%)	1.000 (reference)	1.000 (reference)
C allele	508 (21.4%)	391 (22.6%)	1.075 (0.926–1.248)	1.130 (0.931–1.371)
**rs2120243**				
CC	530 (44.6%)	385 (44.5%)	1.000 (reference)	1.000 (reference)
CA	542 (45.6%)	401 (46.4%)	1.019 (0.847–1.224)	1.062 (0.837–1.347)
AA	117 (9.8%)	79 (9.1%)	0.930 (0.679–1.273)	1.129 (0.753–1.692)
CA + AA	659 (55.4%)	480 (55.5%)	1.003 (0.841–1.196)	1.073 (0.855–1.347)
C allele	1602 (67.4%)	1171 (67.7%)	1.000 (reference)	1.000 (reference)
A allele	776 (32.6%)	559 (32.3%)	0.986 (0.863–1.125)	1.059 (0.893–1.257)

*Adjusted odds ratios (aORs) with their 95% confidence intervals (CIs) were estimated using multiple logistic regression models after controlling for betel quid chewing, cigarette smoking, and alcohol consumption*.

### Combined Effects of Environmental Factors and *PTX3* Gene Polymorphisms on Oral Cancer

To determine the combined effects of environmental factors and *PTX3* gene SNVs on oral cancer susceptibility, we conducted further analysis on 1,397 smokers (Table 4). As presented in Table 4, participants with at least one A allele of rs1840680, one A allele of rs2305619, one C allele of rs3816527, or one A allele of rs2120243 exhibited 12.622-fold (95% CI: 8.725–18.259), 13.171-fold (95% CI: 8.944–19.395), 13.798-fold (95% CI: 9.538–19.960), and 12.794-fold (95% CI: 8.787–18.628) higher risks of oral cancer, respectively, compared with individuals with WT homozygotes who did not chew betel quid.

**Table 4:** Association of the combined effect of *PTX3* gene polymorphisms and betel quid chewing with susceptibility to oral cancer among 1,397 smokers.

**Variable**	**Controls (*n* = 631) (%)**	**Cases (*n* = 766) (%)**	**OR (95% CI)**	***p*-value**	**AOR (95% CI)**	***p*-value**
**rs1840680**						
GG genotype & non-betel quid chewing	208 (33.0%)	53 (6.9%)	1.00 (reference)		1.000 (reference)	
GA or AA genotype or betel quid chewing	312 (49.4%)	356 (46.5%)	**4.478 (3.195–6.277)**	***p*** **<** **0.001**	**4.230 (2.998–5.969)**	***p*** **<** **0.001**
GA or AA genotype with betel quid chewing	111 (17.6%)	357 (46.6%)	**12.622 (8.725–18.259)**	***p*** **<** **0.001**	**10.504 (7.198–15.327)**	***p*** **<** **0.001**
**rs2305619**						
GG genotype & non-betel quid chewing	188 (29.8%)	44 (5.7%)	1.00 (reference)		1.000 (reference)	
GA or AA genotype or betel quid chewing	322 (51.0%)	349 (45.6%)	**4.631 (3.226–6.647)**	***p*** **<** **0.001**	**4.455 (3.081–6.441)**	***p*** **<** **0.001**
GA or AA genotype with betel quid chewing	121 (19.2%)	373 (48.7%)	**13.171 (8.944–19.395)**	***p*** **<** **0.001**	**11.271 (7.586–16.745)**	***p*** **<** **0.001**
**rs3816527**						
AA genotype & non-betel quid chewing	283 (44.8%)	66 (8.6%)	1.00 (reference)		1.000 (reference)	
AC or CC genotype or betel quid chewing	270 (42.8%)	449 (58.6%)	**7.130 (5.243–9.697)**	***p*** **<** **0.001**	**6.652 (4.857–9.112)**	***p*** **<** **0.001**
AC or CC genotype with betel quid chewing	78 (12.4%)	251 (32.8%)	**13.798 (9.538–19.960)**	***p*** **<** **0.001**	**11.827 (8.099–17.271)**	***p*** **<** **0.001**
**rs2120243**						
CC genotype & non-betel quid chewing	194 (30.8%)	54 (7.1%)	1.00 (reference)		1.000 (reference)	
CA or AA genotype or betel quid chewing	339 (53.7%)	363 (47.4%)	**3.847 (2.749–5.383)**	***p*** **<** **0.001**	**3.664 (2.600–5.162)**	***p*** **<** **0.001**
CA or AA genotype with betel quid chewing	98 (15.5%)	349 (45.5%)	**12.794 (8.787–18.628)**	***p*** **<** **0.001**	**10.755 (7.330–15.779)**	***p*** **<** **0.001**

*Adjusted odds ratios (aORs) with their 95% confidence intervals (CIs) were estimated using multiple logistic regression models after controlling for age and alcohol drinking*.

*P-values were adjusted for multiple comparisons by applying the Bonferroni correction*.

*Bold values were considered statistically significant*.

### Effects of Polymorphic Genotypes of *PTX3* on Clinical Status of Oral Cancer

We analyzed the relationship between the combined effect of cigarette smoking and PTX3 variants on oral cancer development. As depicted in Table 5, individuals who possessed the CC allele of rs3816527 and smoked cigarettes were more prone to developing late-stage tumors (stage III/IV: OR, 2.328; 95% CI, 1.078–5.027; *p* = 0.0314) and lymph node metastasis (OR, 2.152; 95% CI, 1.047–4.422; *p* = 0.0371) compared with individuals who were homozygous for the WT allele of rs3816527 (Table 5).

**Table 5:** Genotyping frequency of the *PTX3* rs3816527 polymorphism on clinical status of oral cancer among 766 smokers.

	**Clinical Stage**		**OR (95% CI)**	**AOR (95% CI)^**a**^**
***PTX3*** **rs3816527**	**Stage I/II (*****n*** **=** **379)** ***n*** **(%)**	**Stage III/IV (*****n*** **=** **387)** ***n*** **(%)**		
AA	235 (62.0%)	222 (57.3%)	1.000 (reference)	1.000 (reference)
AC	134 (35.4%)	143 (37.0%)	1.130 (0.838–1.523)	1.111 (0.823–1.500)
CC	10 (2.6%)	22 (5.7%)	**2.328 (1.078–5.027)**^**b**^	**2.424 (1.114–5.273)**^**d**^
	**Tumor size**			
*PTX3* rs3816527	< underline < >T2 (*n* = 436) *n* (%)	> T2 (*n* = 330) *n* (%)		
AA	262 (60.1%)	195 (59.1%)	1.000 (reference)	1.000 (reference)
AC	157 (36.0%)	120 (36.4%)	1.027 (0.760–1.388)	1.034 (0.764–1.400)
CC	17 (3.9%)	15 (4.5%)	1.186 (0.578–2.432)	1.202 (0.582–2.483)
	**Lymph node metastasis**			
*PTX3* rs3816527	No (*n* = 519) *n* (%)	Yes (*n* = 247) *n* (%)		
AA	312 (60.1%)	145 (58.7%)	1.000 (reference)	1.000 (reference)
AC	191 (36.8%)	186 (34.8%)	0.969 (0.702–1.337)	0.950 (0.687–1.312)
CC	16 (3.1%)	16 (6.5%)	**2.152 (1.047**–**4.422)**^**c**^	**2.183 (1.053**–**4.528)**^**e**^
	**Cell differentiation**			
*PTX3* rs3816527	Well (*n* = 111) *n* (%)	Moderate/poor (*n* = 655) *n* (%)		
AA	70 (63.1%)	387 (59.1%)	1.000 (reference)	1.000 (reference)
AC	38 (34.2%)	239 (36.5%)	1.138 (0.743–1.743)	1.117 (0.728–1.713)
CC	3 (2.7%)	29 (4.4%)	1.748 (0.518–5.897)	1.750 (0.515–5.939)

a *Adjustment for the effects of betel quid chewing and alcohol consumption*.

b *p = 0.0314*.

c *p = 0.0371*.

d *p = 0.0256*.

e *p = 0.0359*.

*Bold values were considered statistically significant*.

### Functional Connotation of the *PTX3* rs3816527 Locus

**Figure 1:**
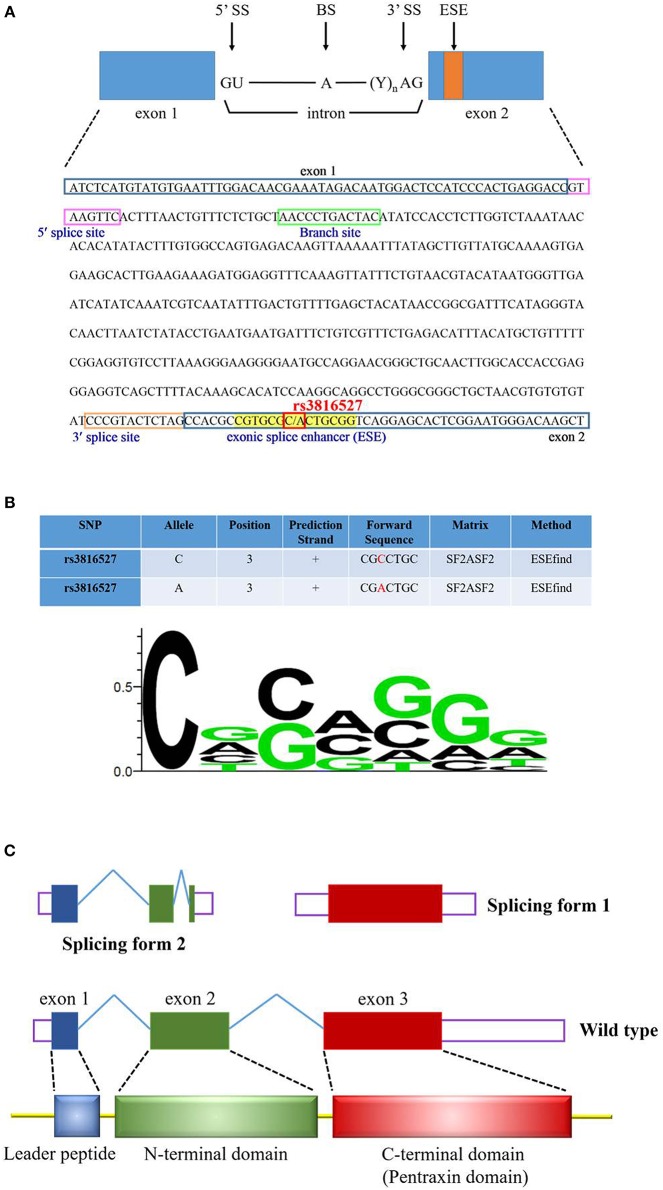
Functional prediction profiling of *PTX3* SNV rs3816527. **(A)** The sequence of *PTX3* is from the NCBI reference sequences (RefSeq) (NM_002852.3). The cis-elements (5′ splice site, 3′ splice site, and branch site) and exonic splice enhancer (ESE) are indicated by various color squares. *PTX3* SNV rs3816527 is located on the ESE sequence. **(B)** The functional prediction of rs3816527 from SNPinfo web server is presented in this Figure. The relative frequency of the nucleotide for the ESE sequence is illustrated by the sequence logo. **(C)** *PTX3* gene consists of three exons. The first exon encodes the leader peptide, and the second and third exons encode N-terminal and C-terminal domains, respectively. Two common splicing forms of *PTX3* are accessed from the NCBI database.

We investigated the functional connotation of the rs3816527 SNV on the *PTX3* gene. The function of *PTX3* rs3816527 was performed using several bioinformatics tools, namely SNPinfo, the NCBI database, ESEfinder, and SWISS-MODEL. Data indicated that *PTX3* rs3816527 was located on the exonic splice enhancer sequence of the *PTX3* gene (Figure 1A). The C allele had stronger enhancement activity than did the A allele and may be more likely to promote the splicing modification of the *PTX3* gene (Figure 1B). Two splicing forms of *PTX3* existed in the NCBI database (Figure 1C).

## Discussion

Head and neck cancers are common worldwide. Approximately 90% of head and neck cancers are squamous cell carcinomas, of which ~50% occur in the oral cavity (38, 39). In south Central Asia, oral cancer is the third most common type of cancer (40). Oral cancer is associated with the use of chronic stimuli such as tobacco smoking, alcohol consumption, and betel quid chewing in particular (3, 4, 41). PTX3 was recognized by proteomics as a critical candidate biomarker for liposarcoma (42), lung cancer (43), prostate cancer (44), and pancreatic cancer (22). For example, a high expression of PTX3 was found in pancreatic cancer cell lines and a direct relationship was found between tumor metastasis and PTX3 expression (22). Moreover, several studies have reported the role of *PTX3* in epithelial cancer progression due to EMT induction (45–48). A low expression of PTX3 has been associated with increased susceptibility to epithelial carcinogenesis (46). Previous studies have demonstrated that PTX3 mediated the induction of the EMT by reducing the expression of E-cadherin, increasing the expression of N-cadherin and vimentin, and promoting the migration of HK-2 cells (45). Moreover, results indicated that the expression of pentraxin family members was significantly associated with the poor prognosis of patients with pancreatic cancer (22). Furthermore, overexpression of PTX3 could promote the proliferation and invasion of cervical cancer *in vitro* and *in vivo* (24). However, few studies have discussed the role of PTX3 in oral cancer. In the present study, the combined effect of environmental factors and *PTX3* polymorphisms considerably increased the risk of oral cancer (Table 4). Moreover, patients with *PTX3* SNV rs3816527 with CC had the highest risk of tumors (Table 5).

The occurrence of cancer stems from the genetic and epigenetic alterations of the basic mechanisms of the normal cell cycle, such as replication control and cell death (49, 50). Most genes in the human genome consist of multiple introns and exons that are modified through splicing to form mature messenger ribonucleic acid and protein products (51, 52). Although alternative splicing (AS) provides cells with protein diversity, studies have found that pathological changes in splicing can contribute to the development of cancers. For instance, mutations and gene expression changes affect the splicing regulatory sequences of key cancer-related genes (53) and the core or accessory components of spliceosome complexes (54–59).

*PTX3* has two splice variants; one contains exon 1 or 2 (length of 453 bp) and the other contains exon 3 (length of 931 bp) (Figure 1C). ESEfinder analysis results revealed that the rs3816527 C allele had superior splicing enhancement activity for the A allele (Figure 1B). In addition, in the protein structure constructed by SWISS-MODEL, PTX3 was split into an N terminus and C terminus after splicing. Although the mechanism of action for this structure remains unclear, a molecular study indicated that the overexpression of PTX3 at the N terminus can considerably inhibit the oncogenic activity of transgenic adenocarcinoma mouse prostate-C2 transfectants, whereas C-terminal overexpression has only a minor effect on tumor growth (60).

We revealed an impact of *PTX3* gene variations on the development of oral cancer; however, there are some limitations in the study. As this study only included a discovery population and not a second independent study to replicate the findings, the associations between *PTX3* variations and oral cancer should be considered preliminary. The other concern is that we failed to exclude the possibility of potential selection bias and since the control group was enrolled among subjects without cancer on a hospital basis. In addition, determining the functional role of PTX3 in the development of oral cancer still requires further investigation.

In conclusion, our results suggest that the allelic effects of *PTX3* SNVs (rs1840680, rs2305619, rs3816527, and rs2120243) enhance the risk and progression of oral cancer in the presence of environmental factors such as tobacco smoking and betel quid chewing. This genetic association was observed most markedly in smokers. These results expose a novel genetic–environmental predisposition to oral cancer carcinogenesis.

## Data Availability

The raw data supporting the conclusions of this manuscript will be made available by the authors, without undue reservation, to any qualified researcher.

## Ethics Statement

The Institutional Review Board of Chung Shan Medical University approved this study (CSMUH No: CS13214-1). All participants provided informed consent.

## Author Contributions

C-MY, C-WL, S-FY, and M-KC contributed to conception, design and critically revised the manuscript. C-MY, S-FY, and M-KC contributed to conception and drafted the manuscript, C-YC, Y-FL, and C-HC contributed to performed experiments and analyzed data.

### Conflict of Interest Statement

The authors declare that the research was conducted in the absence of any commercial or financial relationships that could be construed as a potential conflict of interest.

## Abbreviations

PTX3: long pentraxin 3
OSCC: oral squamous cell carcinoma
OR: odds ratio
SNV: single-nucleotide variation.
